# Male obesity and semen quality: Any association?

**Published:** 2018-04

**Authors:** Obose Rufus, Osaikhuwuomwan James, Aziken Michael

**Affiliations:** *Department of Obstetrics and Gynaecology, College of Medical Sciences, University of Benin, Benin-City, Nigeria.*

**Keywords:** Obesity, Infertility, Semen analysis, Male factor infertility, Body mass index

## Abstract

**Background:**

Infertility as well as obesity are risng global concern. Whilst there is an established association between female obesity and infertility, a similar link is yet to be proven in men.

**Objective:**

To determine the effects of elevated body mass index (BMI) on semen quality among male partners of infertile couples attending an infertility clinic.

**Materials and Methods:**

In this cross-sectional study, 206 men who met the inclusion criteria were recruited for the study. Selected participants were grouped according to their BMI (kg/m^2^): normal BMI (18.5-24.9 kg/m^2^) and elevated BMI (≥25 kg/m^2^). The effect of weight on semen quality was assessed based on sperm count, percentage motility, and morphology.

**Results:**

The number of participants with normal BMI was 110 (53.4%) while those with elevated BMI were 96 (46.6%). Of the participants in elevated BMI group, 52 (25.2%) were overweight and 44 (21.4%) were obese. There was no statistically significant difference in the semen quality as well as the pattern of semen parameter abnormalities between males with normal and elevated BMI (overweight or obese) (p=0.813).

**Conclusion:**

Elevated BMI did not significantly influence semen quality.

## Introduction

Global changes in lifestyle factors and caloric habits are largely responsible for the emerging obesity pandemic. Obesity is a medical condition characterized by accumulation of excess body fat or white adipose tissue, with adverse effect on health and life expectancy (1, 2). Obesity has been associated with several adverse medical conditions including infertility. While there is an established association between female obesity and infertility (anovulation and polycystic ovaries) a similar link is yet to be proven in men (2-4).

Recently, male factor is being implicated as an independent cause of infertility. Male factor infertility constitutes a worldwide problem, especially in Nigeria where most men do not really accept that they contribute to the couple’s infertility. Male factor infertility alone constitutes about 30% and it contributes to another 30% in combination with female factors (4, 5). Some known etiologies of male infertility include genital infections, testicular varicocele, testicular torsion or trauma, previous groin or scrotal surgery, erectile dysfunction, anti-sperm antibodies, chronic and serious systemic illness, hypogonadotropic hypogonadism, gonadal dysgenesis, obstruction of reproductive channels and environmental toxins. Obesity has also been reported to contribute to male infertility albeit there is no consensus (4-8).

Obesity has been postulated to influence male fertility through reduction of semen quality and testosterone level (2). Empirical evidence from a number of studies shows that weight loss, as part of a healthier lifestyle, can help to improve erectile dysfunction. However, evidence to support causality or improved fertility following weight loss intervention programs is still deficient (9 - 12). So it is biologically plausible to suggest that while there are no data to prove that weight loss reverses infertility, optimizing BMI in obese men can normalize sex hormone levels, improve erectile function and possibly semen parameters (11). 

The lack of consensus on the impact of BMI on male fertility or semen parameters against a backdrop of increasing prevalence of obese male partners of infertile couples attending the infertility clinic necessitated the need to evaluate the relationship between elevated BMI and semen parameters at our center. This is with a view to use findings from the study to improve the infertility treatment and counseling.

## Materials and methods

This cross-sectional study was conducted over a 10 month period (September 2013 to June 2014) inclusive of all consenting male partners of infertile couples attending the infertility clinic at the Human Reproduction and Research Programme Unit of the Department of Obstetrics and Gynaecology, University of Benin Teaching Hospital, Benin City, Nigeria.

**Inclusion and exclusion criteria**

Inclusion criteria: All consenting men attending the infertility clinic during the study period with normal descended testes Exclusion criteria: All men with cryptorchidism, testicular varicocele gonadal disease or abnormality, genital infections, previous groin or scrotal surgeries, chronic medical disorders such as uncontrolled hypertension and diabetes, those seropositive for HIV or those on drugs such as steroids and immunosuppressant that may affect spermatogenesis or cause erectile dysfunction and finally those who refused to give consent were excluded. 

**Participants**

The study population comprises male partners of infertile couple attending clinic during the study period. Sample size was determined using the formula for analytic cross sectional study (15), and two hundred and six participants were recruited during the study period. 

Gynaecologic history was taken to evaluate infertility as well as general and physical examination done for all participants. Selected participants had their weight, height, and BMI measured. Semen samples were collected by masturbation after a recommended 3-5 days of abstinence. After collection, specimens were allowed to liquefy at room temperature for 30 min before using for analysis. Microscopy was done for each semen sample. Those with significant pus cells (≥5 pus cells/ high power field) or excessive white blood cells (more than one million/ml) on microscopy which could signify infection were excluded from the study. On microscopic examination, sperm count and the percentage of motility and morphology were objectively evaluated according to the recommendation of World Health Organization (WHO) (13). BMI was categorized according to WHO 2003 BMI classification (14)) and for this study, the study population was categorized into normal BMI (18.5-24.99 kg/m^2^) and elevated BMI ≥25 kg/m^2^ comprising overweight (25-29.99 kg/m^2^) and obesity (≥30 kg/m^2^).

**Definitions relating to semen quality based on WHO manual (13) include**

Normozoospermia: Normal ejaculate

Oligozoospermia: Sperm concentration less than 20 million/ml

Asthenozoospermia: Less than 50% for progressive forward motility

Teratozoospermia: Less than 15% for normal morphology (using the strict criteria)

Oligoasthenoteratozoospermia

Signifies disturbance of all three variables (combinations of only two prefixes may also be used).

Azoospermia: No spermatozoa in the ejaculate.

Aspermia: No ejaculate

Cryptozoospermia: Few spermatozoa recovered after centrifugation

**Outcome measures**

The primary outcome measure was the proportion of patients with elevated BMI with normal and abnormal semen parameters (sperm concentration/count, motility, and morphology) on semen analysis. The secondary outcome measure was their pattern of semen parameter abnormalities (proportion with oligozoospermia, azoospermia, asthenozoospermia, teratozoospermia, and oligoasthenoteratozoospermia).

**Ethical consideration**

Approval for the study was obtained from the Ethical and Research Committee of the University of Benin Teaching Hospital. The study was carefully explained to the patients and informed consent obtained before being recruited into the study. Participation in this research was entirely voluntary. They were counseled that they could opt out of the study at any time if they desired and it will not be held against them in any way, now or in future in their clinical management in the hospital or any of its affiliated institutions.

**Statistical analysis**

Data entry was done using SPSS Statistical Software (Statistical Package for the Social Sciences, version 20.0, SPSS Inc, Chicago, Illinois, USA). Results were presented as frequencies and percentages. Statistical analysis of generated data was calculated using the Chi-square test. Relative risks and Confidence interval employed as appropriate. Statistical significance was set as p≤0.05.

## Results

From a total of 206 men who were recruited for this study, 110 (53.4%) men have normal and 96 (46.6%) has elevated BMI. Of 96 elevated BMI group, 52 (25.2%) were overweight and 44 (21.4%) were obese. Most of the men (60.7%) were in the ≥40 yr age and the majority (65.5%) had tertiary education. Also, there was no statistically significant difference in terms of normal and abnormal semen quality in participants with normal and elevated BMI, (p=0.813) (Table I, II).

Table III shows the classification of semen abnormalities among the participants. The semen abnormalities of participants were grouped into those who had a single defect and those who had combined defects. Asthenozoospermia (39.3%) was noted as the only single defect while Oligoasthenoterato-zoospermia (26.7%) accounted for the majority of the combined defects. Comparison of semen parameters of the study population with BMI, showed that there was no statistically significant difference in the semen parameters (sperm count, motility and morphology) of the study population with normal and elevated BMI (overweight and obesity). Also, a comparison of semen defects amongst BMI groups of study population noted that there was no statistically significant difference in the semen abnormalities (single and combined defects) between men with normal and elevated BMI (overweight and obesity) (Table IV, V).

**Table I:** Socio-demographic characteristics of participants

**Characteristics**		**Frequency n (%) ** **(N= 206)**
Age (yr)		
	20-29	4 (1.9)
	30-39	77 (37.4)
	≥40	125 (60.7)
Educational status		
	Completed primary	9 (4.4)
	Completed secondary	62 (30.1)
	Tertiary	135 (65.5)
Body mass index		
	Normal (18.5-24.9 kg/m2)	110 (53.4)
	Elevated (≥25 kg/m2)	96 (46.6)

Data presented as n (%)

**Table II:** Semen characteristics of study population

**Semen quality**	**Normal BMI (n= 110)**	**Elevated BMI F(n= 96)**	**p-value**
Normal	17 (15.5)	16 (16.7)	0.813
Abnormal	93 (84.5)	80 (83.3)	

Data presented as n (%).

BMI: Body mass index; Normal BMI: 18.5-24.9 kg/m^2^, Elevated (≥25 kg/m^2^). chi-squared (χ)^2^ test analysis used

**Table III:** Classification of semen abnormalities

**Semen abnormalities**		**Frequency (%)**
Single defects		
	Oligozoospermia	0 (0.0)
	Asthenozoospermia	81 (39.3)
	Teratozoospermia	0 (0.0)
Combined defects		
	Astheno/oligozoospermia	20 (9.7)
	Oligo/teratozoospermia	6 (2.9)
	Astheno/teratozoospermia	0 (0.0)
	Oligo/Astheno/teratozoospermia	55 (26.7)
	Azoospermia	11 (5.3)

Data presented as n (%).

**Table IV:** Comparison of semen parameters of the study population with BMI

**Semen parameters**		**Body mass index**			**Relative risk**	**Confidence interval**	**p-value**
		**Normal**	**Overweight**	**Obesity**			
Sperm count							
	Normozoospermia	66 (60.0)	28 (53.9)	20 (45.5)	1.800	0.889–3.645	0.102
	Oligozoospermia	38 (34.6)	21 (40.4)	22 (50.0)	0.528	0.260–1.073	0.078
	Azoospermia	6 (5.5)	3 (5.8)	2 (4.6)	1.212	0.235–6.245	0.819
Motility							
	Normal	25 (22.7)	10 (19.2)	7 (15.9)	1.555	0.618–3.912	0.349
	Asthenozoospermia	79 (71.8)	39 (75.0)	35 (79.6)	0.655	0.282–1.521	0.325
	Azoospermia	6 (5.5)	3 (5.8)	2 (4.6)	1.212	0.235–6.245	0.819
Morphology							
	Normal	92 (83.6)	46 (88.5)	38 (86.4)	0.807	0.297–2.190	0.674
	Teratozoospermia	12 (10.9)	3 (5.8)	4 (9.1)	1.224	0.373–4.024	0.739
	Azoospermia	6 (5.5)	3 (5.8)	2 (4.6)	1.212	0.235–6.245	0.819

BMI: Body mass index (Normal: 18.5-24.9 kg/m^2^, Overweight: 25-29.99 kg/m^2^, Obesity: ≥30 kg/m^2^). chi-squared (χ)^2^ test analysis used

* Data presented as n (%)

**Table V:** Comparison of semen defects amongst BMI groups of the study population

**Semen abnormalities**		**Body mass index**			**Relative risk **		**Confidence interval **	**p-value**
		**Normal **	**Overweight**	**Obesity**				
Single defects								
	Oligozoospermia	0 (0.0)	0 (0.0)					
	Asthenozoospermia	49 (44.6)	18 (34.6)	14 (31.8)	1.721	0.823–3.599		0.149
	Teratozoospermia	0 (0.0)	0 (0.0)					
Combined defects								
	Asthenooligozoospermia	7 (6.4)	6 (11.5)	7 (15.9)	0.359	0.118–1.093		0.071
	Oligoteratozoospermia	4 (3.6)	1 (1.9)	1 (2.3)	1.660	0.180–15.277		0.654
	Asthenoteratozoospermia	0 (0.0)	0 (0.0)	0 (0.0)	–	–		–
	Oligoasthenoteratozoospermia	27 (24.6)	16 (30.8)	12 (27.3)	0.867	0.393–1.917		0.725
	Azoospermia	6 (5.5)	3 (5.8)	2 (4.6)	1.212	0.235–6.245		0.819

BMI: Body mass index (Normal: 18.5-24.9 kg/m^2^, Overweight: 25-29.99 kg/m^2^, Obesity: ≥30 kg/m^2^. chi-squared (χ) 2 test analysis used.

* Data presented as n (%).

## Discussion

The rising trend of obesity amidst global modernization and industrialization as well as environmental and lifestyle changes with its attendant health hazard are cause for global concern. Whilst obesity has been associated with several adverse medical outcomes its role in male infertility has not been clearly defined, with conflicting reports from different authors (6, 7, 10). 

This study demonstrated that semen parameters (count, motility, and morphology) were comparable among the groups irrespective of BMI: whether normal, overweight or obese. Thus we can posit that no significant association exists between elevated BMI and semen quality amongst infertile men in this setting. A imilar finding was reported by Mac Donald and co-workers in a systematic review of a meta-analysis of the impact of body mass index on semen parameters which found no association between BMI and semen parameters (16). Chavarro and colleagues also in a cross-sectional study of body mass index in relation to semen quality, sperm DNA integrity and serum reproductive hormone levels did not observe any association between BMI and semen parameters (17). There are other studies which shared similar finding (18, 19). However, on the contrary, some studies noted a negative association between BMI and semen parameters with variation in reports on the particular semen parameter affected (6, 20-22). A cross-sectional study of 1,558 Danish military male recruits to evaluate body mass index in relation to semen quality and reproductive hormones noted that men with elevated BMI (>25 kg/m^2^) had a lower sperm concentration and total sperm count than those with normal BMI (22). Hammoud *et al* in a retrospective study of male obesity and alteration in sperm parameters noted also that male obesity is associated with increased incidence of low sperm concentration and low progressive motility (6). However nonuniformity in cut off values for BMI used in these studies may contribute to variations in results obtained. It is relevant to note that these studies which showed a negative association between BMI and semen parameters recorded no effect on sperm morphology. Although not influenced by BMI, this study showed motility abnormalities (Asthenozoospermia) to be the most common single disorder among the study population with 39.3%. A similar finding was noted in a study by Akinola *and *co-workerswith motility abnormality of 24.9%. Sperm motility has been reported to have a much stronger relationship to both percentages of pregnancy and conception rate when compared to sperm concentration (5), albeit sperm morphology appears to be more stable.

Although this study has shown that elevated body mass index does not appear to have any effect on semen quality. However, a larger sample size, possibly multicentred prospective study with standardization of semen analysis technique may be necessary in order to arrive at a logical and representative conclusion of the effect of BMI on semen quality in our setting.

Despite the limitations we can conclude from this study that no considerable difference exists in the semen quality as well as the pattern of semen abnormalities between male partners of infertile couples with normal and elevated BMI (overweight or obese), thus increasing the existing conflict of evidence with regard to male infertility and obesity. 

## Conclusion

There is no controversy that overweight and obesity have become major health concerns worldwide and the benefits of weight control cannot be over emphasized. Male partners of infertile couples with elevated BMI seeking treatment can be reassured that their BMI may not adversely affect their semen quality as well as their quest for conception but overall obesity is discouraged for healthy living.
